# Mediating effect of cardiorespiratory fitness on the relationship between inspiratory muscle strength and quality of life in people on hemodialysis

**DOI:** 10.1590/2175-8239-JBN-2025-0175en

**Published:** 2026-03-20

**Authors:** Júlia Garcia de Castro Pereira, Inara Caroline Marcelino Martins, Cecília Alves Macedo, Joyce Noelly Vitor Santos, Elisângela Andrade Assis Madeira, Vanessa Gomes Brandão Rodrigues, Maria Cecília Sales Mendes Prates, Frederico Lopes Alves, Emílio Henrique Barroso Maciel, Vanessa Pereira Lima, Henrique Silveira Costa, Vanessa Amaral Mendonça, Pedro Henrique Scheidt Figueiredo

**Affiliations:** 1 Universidade Federal dos Vales do Jequitinhonha e Mucuri, Programa de Pós-Graduação em Reabilitação e Desempenho Funcional, Diamantina, MG, Brazil.; 2 Universidade Federal dos Vales do Jequitinhonha e Mucuri, Departamento de Fisioterapia, Diamantina, MG, Brazil.; 3 Universidade Federal dos Vales do Jequitinhonha e Mucuri, Programa de Pós-Graduação em Ciências da Saúde, Diamantina, MG, Brazil.; 4 Santa Casa de Caridade de Diamantina, Setor de Hemodiálise, Diamantina, MG, Brazil.

**Keywords:** Respiratory Muscles, Exercise Tolerance, Muscle Strength, Quality of Life, Hemodialysis, Sustainable Development, ODS-3.

## Abstract

**Introduction:**

Patients undergoing hemodialysis often exhibit inspiratory muscle weakness, which has been associated with a reduced health-related quality of life (HRQoL). However, the extent to which this relationship is mediated by cardiorespiratory fitness remains unclear.

**Objective:**

To evaluate the mediating effect of cardiorespiratory fitness on the association between inspiratory muscle strength and HRQoL in hemodialysis patients.

**Methods:**

Cross-sectional study in which hemodialysis patients underwent assessment of inspiratory muscle strength by measuring Maximum Inspiratory Pressure (MIP), cardiorespiratory fitness by the Incremental Shuttle Walk Test (ISWT), and HRQoL by measuring the physical, mental, and general health component summary scores of the Kidney Disease Quality of Life questionnaire (KDQOL-SF). Simple mediation analyses were performed using the PROCESS macro (version 3.5) for SPSS, with HRQoL as the dependent variable, MIP as the independent variable, and cardiorespiratory fitness as the mediating variable.

**Results:**

64 individuals, 67.2% male, with a mean age of 48.1 years (95%CI 44.3–51.9), were evaluated. Cardiorespiratory fitness exerted a partial mediating effect on the association between MIP and PCS (β = 0.14; 95%CI: 0.02–0.27), resulting in a 6% reduction in PCS variance explained by MIP (6% of R2). The mediating proportion was 32.5%. There was no association between MIP and the mental component, and cardiorespiratory fitness did not mediate the relationship between MIP and the general component of HRQoL (β = 0.13; p = 0.350).

**Conclusion:**

In the sample studied, the association between inspiratory muscle strength and the physical aspects of HRQoL is partially mediated by cardiorespiratory fitness. However, this mediating effect was not observed for the mental or general aspects of HRQoL.

## Introduction

Chronic Kidney Disease (CKD) is characterized by a progressive and irreversible decline in renal function resulting from kidney injury lasting more than three months, with negative impacts on health^1^. As the disease progresses, the renal system becomes unable to maintain homeostasis. At this stage, renal replacement therapy is indicated^1^, with hemodialysis being the most widely used modality^2^. According to the 2023 Brazilian Dialysis Census^3^, the average number of patients undergoing dialysis was 157,357, with prevalence and incidence rates of 771 and 251 cases per million population, respectively.

Although hemodialysis is essential for symptom control and improved survival, both the treatment itself and CKD can alter muscle structure and function, resulting in reduced cardiorespiratory fitness^4,5^. Once present, reduced cardiorespiratory fitness may contribute to limitations in activities requiring physical effort, affecting activities of daily living and emotional well-being, and ultimately leading to a decline in health-related quality of life (HRQoL)^6^. Additionally, impaired cardiorespiratory fitness is associated with a worse prognosis in this population^7,8^.

Alterations in muscle structure and function in hemodialysis patients may also impact inspiratory muscle performance, as evidenced by a reduction in maximal inspiratory pressure (MIP)^9,10^. Reduced MIP has been identified as an independent predictor of impaired cardiorespiratory fitness in this population^11^ and is also associated with declines in HRQoL^12^. The relationship between inspiratory muscle strength and HRQoL has further been inferred from the positive outcomes of targeted inspiratory muscle training^11,13,14^.

Therefore, the association between MIP and cardiorespiratory fitness in individuals with CKD undergoing hemodialysis has already been described in the literature, as has the association between MIP and HRQoL. However, it remains unclear whether inspiratory muscle strength exerts a direct effect on HRQoL or whether this association reflects solely an indirect influence mediated by reduced cardiorespiratory fitness. Clarifying this question may contribute to a deeper understanding of the mechanisms underlying the relationship between inspiratory muscle strength and HRQoL. The present study aimed to evaluate the mediating effect of cardiorespiratory fitness on the association between inspiratory muscle strength and HRQoL in individuals undergoing hemodialysis.

## Methods

### Study Design

This was a cross-sectional study conducted at the Hemodialysis Unit of *Santa Casa de Caridade de Diamantina* (MG, Brazil), in partnership with the Cardiovascular Rehabilitation Laboratory (LABCAR) at the *Universidade Federal dos Vales do Jequitinhonha e Mucuri* (UFVJM, Diamantina, MG, Brazil). The study was approved by the Institutional Ethics Committee (CAAE 60169822.10000.5108), and all participants provided written informed consent prior to study enrollment.

### Participants

From May to August 2023, individuals with CKD undergoing hemodialysis for more than three months, three times per week, aged 18 years or older, and of both sexes were evaluated. Individuals were excluded if they presented resting systolic and/or diastolic blood pressure greater than 180 and/or 110 mmHg, respectively^15^; severe anemia (hemoglobin < 8 g/dL)^16^; unstable angina; complex ventricular arrhythmias; severe metabolic disease; acute myocardial infarction within the previous month; acute medical conditions; aortic aneurysm; severe aortic stenosis; respiratory, neurological, or musculoskeletal impairments contraindicating physical activity; or inability to complete the assessment protocol.

Sample size calculation was performed using G*Power software, version 3.1 (Franz Faul, University of Kiel, Germany). An estimated sample of 50 participants was derived, considering an adjusted R^2^ of 0.23^12^ between MIP and the physical component summary of the Kidney Disease Quality of Life questionnaire (KDQOL-SF)^17^, a statistical power of 80%, an alpha error of 5%, and five predictors.

### Procedures

Assessments were conducted within a single week, immediately before hemodialysis sessions, in the following sequence^18^: immediately before the first weekly session – inspiratory muscle strength; immediately before the second weekly session – HRQoL; and immediately before the third weekly session – cardiorespiratory fitness.

### Inspiratory muscle strength

Inspiratory muscle strength was assessed by measuring MIP using a calibrated analog vacuum manometer (MV-300/300, Ger-Ar, São Paulo, Brazil), with a measurement range of -300 to 300 cmH_2_O and an accuracy of 10 cmH_2_O. The device was connected to the participant’s mouth via a 14-cm plastic tube with a 2-cm diameter, attached to a mouthpiece containing a 2-mm leak on its upper surface to compensate for the pressure changes induced by oropharyngeal muscles^19^.

For the assessment, participants remained seated at rest for five minutes, with their backs supported by the chair, feet flat on the floor, and hips and knees positioned at 90°. They were instructed to perform a maximal inspiratory effort from residual volume, with the nostrils occluded by a nasal clip. Inspiratory maneuvers were repeated for familiarization, followed by three technically satisfactory measurements with a 60-second rest between each. Measurements were considered satisfactory when they varied by less than 10%, showed no air leakage, and were sustained for at least one second. The highest value was recorded for analysis, provided it was not obtained during the last maneuver. Standardized verbal encouragement was provided during maximal inspiratory efforts^12,20^. Inspiratory muscle weakness was defined as MIP values below the lower limits of normality, based on reference values for healthy Brazilian adults^21^.

### Cardiorespiratory fitness

Cardiorespiratory fitness was assessed using the incremental shuttle walk test (ISWT), conducted in a 10-meter corridor marked by two cones^22^. The 12-level protocol was applied. Participants were instructed to walk according to the pace dictated by the audio signal and did not receive any verbal encouragement from the assessors. The test was terminated when the participant failed to reach the minimum speed required at a given level on two consecutive occasions^11,23^.

### Health-related quality of life (HRQol)

HRQoL was assessed using the KDQOL-SF^17^, a disease-specific questionnaire for individuals with CKD that has been culturally adapted and validated for the Brazilian population^24^. This instrument consists of 24 multidimensional questions, including 43 kidney disease–specific items, 36 generic items, and one item assessing self-rated health status. The KDQOL-SF items are grouped into 19 domains, including 11 dialysis-specific domains and 8 generic domains. Domain scores range from 0 (worst health status) to 100 (best health status). Generic domains were used to calculate the physical component summary (PCS) and the mental component summary (MCS). The dialysis-specific domains were used to calculate the general component summary (GCS)^17^. The component summary scores were the variables analyzed in this study.

### Statistical Analysis

Data analysis was performed using SPSS software, version 25.0. Categorical variables are presented as absolute and relative frequencies, and continuous variables are presented as mean ± standard deviation. Correlation analysis was carried out using the Pearson or Spearman correlation coefficients, according to the results of the normality assessment by the Kolmogorov–Smirnov test. Correlations were classified as very weak when r < 0.31 or > −0.31; weak when r ranged from 0.31 to 0.50 or from −0.50 to −0.31; moderate when r ranged from 0.51 to 0.70 or from −0.70 to −0.51; and strong when r > 0.71 or < −0.71^25^. Statistical significance was set at p < 0.05.

To test the hypotheses regarding the mediating effects of cardiorespiratory fitness on the relationship between MIP and HRQoL, the PROCESS macro (version 3.5) for SPSS was used, with a bootstrapping procedure of 5,000 samples to estimate 95% confidence intervals (95%CI). In simple mediation models, relationships among three variables are examined: the independent variable (MIP), the mediator (cardiorespiratory fitness), and the dependent variable (HRQoL). Thus, the effect of the independent variable on the mediator (MIP → cardiorespiratory fitness), the effect of the mediator on the dependent variable (cardio­respiratory fitness → HRQoL), and the total, direct, and indirect effects of the independent variable (MIP) on the dependent variable (HRQoL) are assessed.

The total effect refers to the overall impact of the independent variable on the dependent variable (MIP → HRQoL). The direct effect represents the impact of the independent variable on the dependent variable when the mediator is included in the model (MIP → HRQoL, adjusted for cardiorespiratory fitness). The indirect effect represents the portion of the association between the independent and dependent variables that operates through the mediator (MIP → cardiorespiratory fitness → HRQoL). A statistically significant indirect effect indicates that cardiorespiratory fitness mediates the relationship between MIP and HRQoL.

The mediating effect may be either complete or partial. When mediation is complete, the direct effect becomes nonsignificant after the mediator (cardiorespiratory fitness) is included. In partial mediation, the magnitude of the regression decreases, and the direct effect coefficient is reduced relative to the total effect, although it remains statistically significant (p < 0.05). Accordingly, cardiorespiratory fitness exerts a partial mediating effect on the association between MIP and HRQoL. In other words, the relationship between MIP and HRQoL is partially mediated by cardiorespiratory fitness^26^. The mediation proportion, which represents the strength of mediation or the extent to which cardiorespiratory fitness explains the association between MIP and HRQoL, can also be calculated^26^. The mediation proportion was calculated by dividing the indirect effect by the total effect^26^.

## Results

**Figure 1:**
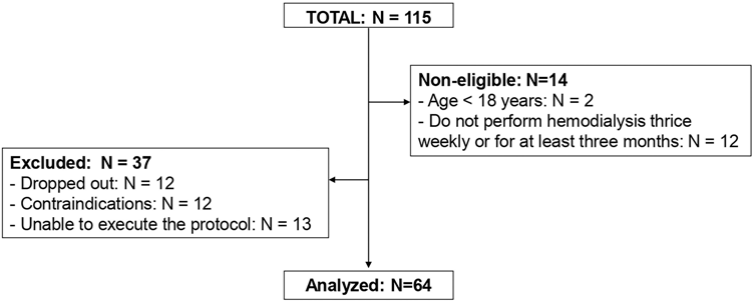
Flowchart.

Of the 115 patients on hemodialysis screened, 101 were considered eligible, and 64 individuals were included in the study (Figure 1). Sample characteristics are shown in Table 1. Participants were predominantly male, with a mean age of 48.1 years (95%CI 44.3–51.9) and eutrophic. The most prevalent comorbidity was systemic arterial hypertension (82.8%). Most participants were using erythropoietin (90.6%). Inspiratory muscle weakness was observed in 21 individuals (32.8%).

**Table 1:** SAMPLE CHARACTERISTICS (N = **64**)

Variables	Value
Sex, n (%)	
Male	43 (67.2)
Female	21 (32.8)
Age (years)	48.1 ± 15.1
BMI (Kg/m2)	24.1 ± 5.1
Treatment duration (years)	4.7 ± 4.2
Biochemistry	
Kt/V	1.5 ± 0.3
Hemoglobin (mg/dL)	10.6 ± 1.9
Hematocrit (%)	33.8 ± 4.6
Inspiratory Muscle Strength	
MIP (cmH2O)	-90.1 ± 26.3
Inspiratory muscle weakness, n (%)	21 (32.8%)
ISWT	
Distance (m)	419.7 ± 224.4

Abbreviations – BMI: Body Mass Index; Kt/V: Hemodialysis Treatment Effectiveness Index; MIP: Maximum Inspiratory Pressure; ISWT: Incremental Shuttle Walk Test.

Note – Data presented as mean ± standard deviation or n (%).

Table 2 presents the HRQoL domain scores. The most impaired components were physical and mental. A significant weak correlation was observed between MIP and both the PCS and GCS of the KDQOL-SF (r = 0.40, p = 0.001; r = 0.43, p < 0.001, respectively). No significant correlation was found between MIP and MCS (r = 0.11, p = 0.38). ISWT distance showed a significant weak correlation with the PCS (r = 0.38, p = 0.002) and a moderate correlation with MIP (r = 0.58, p < 0.001). No significant correlations were found between ISWT distance and GCS or MCS (r = 0.27, p = 0.29; r = 0.98, p = 0.44, respectively). Based on the observed correlations, mediation analyses were conducted using only PCS and GCS as dependent variables.

**Table 2:** DOMAINS OF HEALTH-RELATED QUALITY OF LIFE USING THE KDQOL-SF QUESTIONNAIRE

Variables	Value
KDQOL-SF	
Specific Dimension	
Symptoms	85.1 ± 13.6
Effects of CKD	77.2 ± 17.8
Burden of CKD	44.7 ± 30.8
Work	26.3 ± 39.7
Cognition	90.2 ± 15.0
Social Interaction	88.0 ± 22.2
Sexual Function	50.3 ± 49.5
Sleep	76.1 ± 24.9
Social Support	84.1 ± 29.1
Encouragement by the Health Team	83.3 ± 30.4
Satisfaction with Treatment	80.9 ± 20.3
GCS	71.5 ± 12.1
Generic Dimension	
Functional Capacity	75.2 ± 26.4
Physical Function	62.0 ± 33.3
Pain	73.6 ± 26.1
General Health Status	55.1 ± 27.1
Emotional Well-Being	66.6 ± 40.2
Emotional Role	81.3 ± 22.8
Social Function	88.0 ± 22.2
Energy/Fatigue	70.8 ± 25.5
PCS	46.4 ± 10.3
MCS	44.9 ± 20.7

Abbreviations – KDQOL-SF: Kidney Disease Quality of Life; CKD: Chronic Kidney Disease; PCS: Physical Component Summary; MCS: Mental Component Summary; GCS: General Component Summary.

Note – Data presented as mean ± standard deviation.

The results of the mediation analyses between MIP and the PCS are shown in Supplementary File 1 and Table 3. A significant partial mediating effect of cardiorespiratory fitness on the association between MIP and PCS was observed (β = 0.14; 95%CI: 0.02–0.27). The inclusion of cardiorespiratory fitness as the mediating variable resulted in a 6% reduction in the variance of PCS scores explained by MIP (6% reduction in R^2^). The mediation proportion was 32.5%. No mediating effect was found for the association between MIP and GCS (β = 0.13; p = 0.350), as shown in Supplementary File 2 and Table 4.

**Table 3:** MEDIATION ANALYSIS OF CARDIORESPIRATORY FITNESS FOR THE RELATIONSHIP BETWEEN MAXIMAL INSPIRATORY PRESSURE AND HEALTH-RELATED QUALITY OF LIFE (PHYSICAL COMPONENT SUMMARY)

Effects of variables	R^2^	β	p
Effect of the mediating variable on the dependent variable	0.17	0.28	0.033
Effect of the independent variable on the mediating variable	0.24	0.50	< 0.001
Total effect of the independent variable on the dependent variable	0.19	0.43	< 0.001
Direct effect of the independent variable on the dependent variable (including the mediating variable)	0.25	0.29	0.028
	β	95%CI	p
Indirect effect of the independent variable on the dependent variable	0.14	0.02–0.27	0.028

Notes – Independent variable: maximal inspiratory pressure; Dependent variable: physical component summary of health-related quality of life; Mediator variable: cardiorespiratory fitness; R^2^: coefficient of determination; β: standardized coefficient; 95%CI: 95% confidence interval.

**Table 4:** MEDIATION ANALYSIS OF CARDIORESPIRATORY FITNESS FOR THE RELATIONSHIP BETWEEN MAXIMAL INSPIRATORY PRESSURE AND HEALTH-RELATED QUALITY OF LIFE (GENERAL COMPONENT SUMMARY)

Effects of variables	R^2^	β	p
Effect of the mediating variable on the dependent variable	0.07	0.13	0.350
Effect of the independent variable on the mediating variable	0.16	0.50	< 0.001
Total effect of the independent variable on the dependent variable	0.15	0.40	< 0.001
Direct effect of the independent variable on the dependent variable (including the mediating variable)	0.18	0.34	0.013
	β	95%CI	p
Indirect effect of the independent variable on the dependent variable	–	–	–

Notes – Independent variable: maximal inspiratory pressure; Dependent variable: general component summary of health-related quality of life; Mediator variable: cardiorespiratory fitness; R^2^: coefficient of determination; β: standardized coefficient; 95%CI: 95% confidence interval.

## Discussion

The present study aimed to evaluate the mediating effect of cardiorespiratory fitness on the association between MIP and HRQoL. To the best of our knowledge, this is the first study to address this question specifically, and the main findings were as follows: (1) the presence of a direct association between MIP and HRQoL; and (2) that the association between MIP and the physical component of HRQoL is partially mediated by cardiorespiratory fitness. These findings have important clinical implications, as they highlight the influence of MIP on HRQoL and the mediating role of cardiorespiratory fitness in this relationship.

It is well established in the literature that cardiorespiratory fitness is impaired in individuals undergoing hemodialysis due to the combined effects of the natural progression of the disease, the dialysis procedure itself, and lifestyle changes imposed by the treatment. These factors lead to restrictions in social participation and negatively affect HRQoL^4,5,6,27^. Consequently, limitations in performing activities of daily living and restrictions in social and leisure participation—driven by the combination of clinical signs and symptoms—contribute to declines in HRQoL^27^. Our findings support this evidence, as we also identified an association between cardiorespiratory fitness and HRQoL, particularly in the physical components of the KDQOL-SF.

Our initial analysis revealed a significant association between MIP and both the PCS and GCS of the KDQOL-SF, highlighting the impact of inspiratory muscle strength on HRQoL in patients undergoing hemodialysis. This result is in line with the findings of Vieira *et al*. (2020)^12^, who identified MIP as a predictor of HRQoL independent of hemodialysis duration, age, associated comorbidities, and lower-limb muscle strength.

In addition to its association with HRQoL, MIP has been identified as a determinant of cardiorespiratory fitness in patients on hemodialysis^10^. Thus, inspiratory muscle weakness may negatively impact cardiorespiratory fitness, contributing to declines in HRQoL^11,12^. In the present study, the association between MIP and HRQoL was found to be mediated by cardiorespiratory fitness. This finding suggests that reduced inspiratory muscle strength may impair ventilatory capacity, leading to decreased cardiorespiratory fitness and, conse­quently, affecting the physical aspects of HRQoL.

However, a key finding of the present study was the association between MIP and the general aspects of HRQoL, independent of cardiorespiratory fitness. This direct association suggests that MIP influences HRQoL through pathways that do not rely exclusively on cardiorespiratory fitness. Although relationships among inspiratory muscle strength, cardiorespiratory fitness, and HRQoL have been previously described in individuals undergoing hemodialysis^10,12,28^, no studies have examined whether MIP is associated with HRQoL independently of cardiorespiratory fitness, which limits direct comparisons with our findings.

HRQoL in hemodialysis patients is multi­dimensional^29^ and is strongly influenced by symptom burden^30^. Campos *et al*. (2024)^31^ demonstrated that limiting symptoms—such as dyspnea, fatigue, and weakness—were reported as key determinants of HRQoL in a sample of individuals on hemodialysis. Inspiratory muscle weakness may reflect systemic alterations in muscle structure and function, which are common in this population and may indicate poorer nutritional status or reduced muscle reserves^32^. These conditions are well known to be associated with symptom manifestation and poorer perceived quality of life^29^.

Furthermore, considering that increased respiratory muscle workload is a mechanism associated with the onset of dyspnea^33^ and fatigue^34^, reduced MIP would be expected to predispose individuals to greater respiratory effort and more pronounced symptom manifestation, thereby limiting their ability to perform activities of daily living. These physiological mechanisms are consistent with previous observational findings, such as those reported by Vieira *et al*. (2020)^12^, who identified a significant correlation between MIP and the “symptoms” domain of the KDQOL (r = 0.45; p < 0.001). Nevertheless, the mechanisms underlying the direct relationship between inspiratory muscle strength and the general aspects of HRQoL in this population require further elucidation in future studies.

The association among MIP, cardiorespiratory fitness, and HRQoL is also indirectly supported by evidence showing that inspiratory muscle training improves respiratory muscle strength, cardiorespiratory fitness, and HRQoL in this population^11,12,13^. Therefore, it can be inferred that increases in inspiratory muscle strength exert a direct positive effect on HRQoL and an additional indirect effect mediated through improvements in cardiorespiratory fitness^27,35^.

In addition to the benefits of inspiratory muscle training, studies have proved that traditional exercise performed during hemodialysis sessions improves both cardiorespiratory fitness and inspiratory muscle strength and function^32^. This finding suggests that traditional exercise-based interventions that enhance inspiratory muscle strength and cardiorespiratory fitness may also positively influence HRQoL in this population.

The present study, using a rigorous methodology, demonstrated the mediating role of exercise capacity in the relationship between MIP and the physical aspects of HRQoL, as well as the association between MIP and the general aspects of HRQoL independent of exercise capacity. These findings are clinically relevant, as they highlight the importance of preventive and rehabilitative strategies to maintain or improve inspiratory muscle function through nutritional interventions and targeted inspiratory muscle training. Such strategies may mitigate declines in HRQoL or even promote improvements among patients undergoing hemodialysis. However, interventional studies are still needed to confirm this hypothesis.

As a limitation, the sample in this study consisted predominantly of younger individuals with fewer comorbidities compared with other cohorts. Therefore, the generalization of these findings to populations with different clinical and demographic characteristics should be interpreted with caution.

In conclusion, among individuals undergoing hemodialysis, the association between inspiratory muscle strength and the physical aspects of HRQoL is partially mediated by cardiorespiratory fitness. However, this mediating effect was not observed for the mental or general aspects of HRQoL.

## Data Availability

The datasets generated and/or analyzed during this study are available upon reasonable request to the corresponding author.

## Supplementary Material

The following online material is available for this article:

Supplementary file 1 - Mediating effect of cardiorespiratory fitness on the relationship between MIP and the physical component summary of health-related quality of life.

Supplementary file 2 - Mediating effect of cardiorespiratory fitness on the relationship between MIP and the general component summary of health-related quality of life.
