# Supplementary material for: Mediating effect of cardiorespiratory fitness on the relationship between inspiratory muscle strength and quality of life in people on hemodialysis
Source: J Bras Nefrol. 2026 Mar 20;48(2):e20250175. doi: 10.1590/2175-8239-JBN-2025-0175en (PMC13004158; doi:10.1590/2175-8239-JBN-2025-0175en)
Supplement: Supplementary file 1 [file 2175-8239-jbn-48-2-e20250175-suppl1.pdf]

Braz. J. Nephrol.
https://doi.org/10.1590/2175-8239-JBN-2025-0175en

Supplementary Material to "Mediating effect of cardiorespiratory fitness on the relationship between inspiratory muscle strength and quality of life in people on hemodialysis"

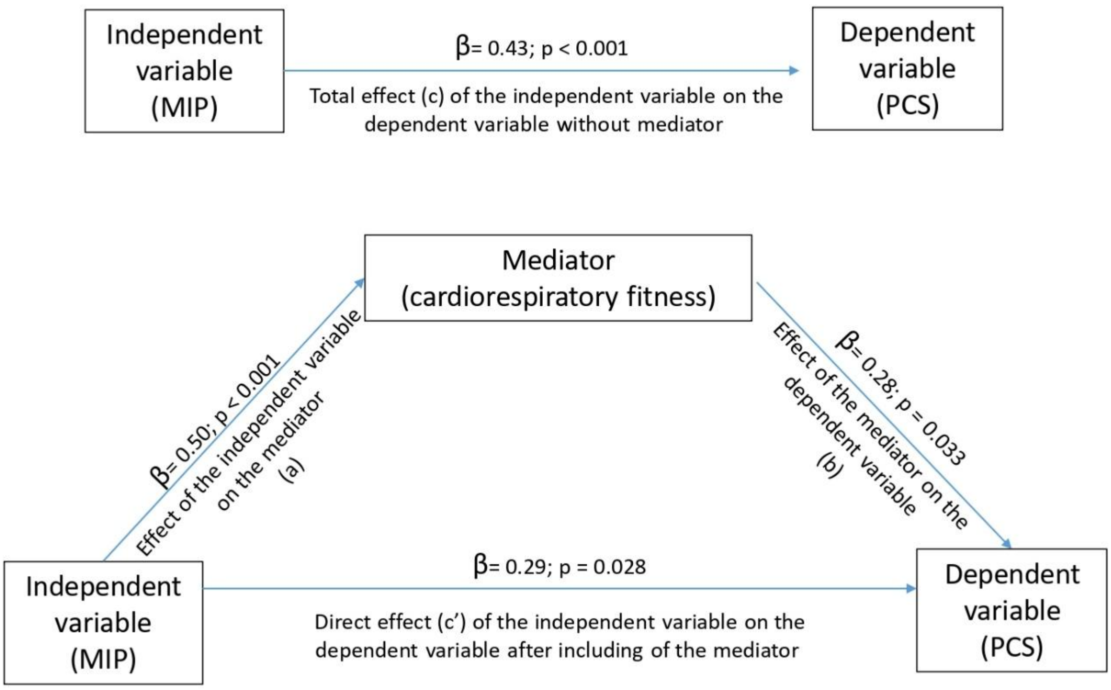

Abbreviations – MIP: Maximum Inspiratory Pressure; ISWT: Incremental Shuttle Walk Test; PCS: Physical Component Summarized.

Supplementary file 1 - Mediating effect of cardiorespiratory fitness on the relationship between MIP and the physical component summarized of health-related quality of life.
