# Supplementary material for: Mediating effect of cardiorespiratory fitness on the relationship between inspiratory muscle strength and quality of life in people on hemodialysis
Source: J Bras Nefrol. 2026 Mar 20;48(2):e20250175. doi: 10.1590/2175-8239-JBN-2025-0175en (PMC13004158; doi:10.1590/2175-8239-JBN-2025-0175en)
Supplement: Supplementary file 3 [file 2175-8239-jbn-48-2-e20250175-suppl3.pdf]

Braz. J. Nephrol.
https://doi.org/10.1590/2175-8239-JBN-2025-0175pt

Material Suplementar para "Efeito mediador da aptidão cardiorrespiratória sobre a relação entre a força muscular inspiratória e a qualidade de vida em pessoas em hemodiálise"

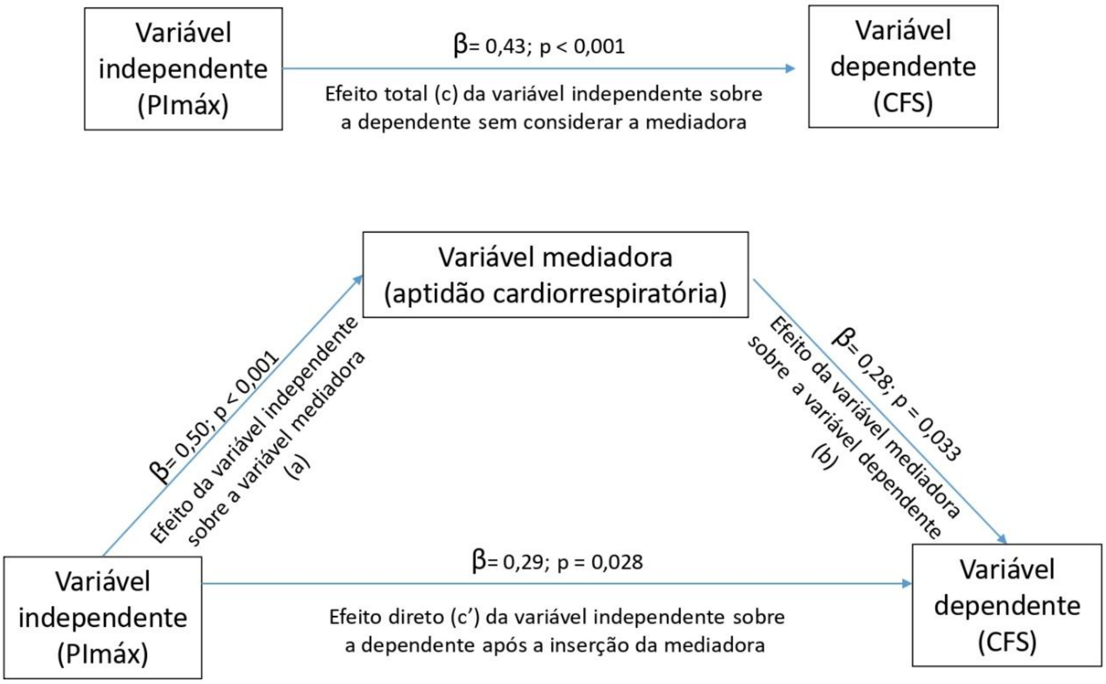

Abreviaturas – PImáx: Pressão Inspiratória Máxima; ISWT: *Incremental Shuttle Walk Test*; CFS: Componente Físico Sumarizado.

Arquivo suplementar 1 - Efeito mediador da aptidão cardiorrespiratória na relação entre PImáx e componente físico sumarizado da qualidade de vida relacionada à saúde.
