# Supplementary material for: Mediating effect of cardiorespiratory fitness on the relationship between inspiratory muscle strength and quality of life in people on hemodialysis
Source: J Bras Nefrol. 2026 Mar 20;48(2):e20250175. doi: 10.1590/2175-8239-JBN-2025-0175en (PMC13004158; doi:10.1590/2175-8239-JBN-2025-0175en)
Supplement: Translation

# Translation: Efeito mediador da aptidão cardiorrespiratória sobre a relação entre força muscular inspiratória e qualidade de vida em pessoas em hemodiálise

**DOI:** 10.1590/2175-8239-JBN-2025-0175pt

**Authors:** Júlia Garcia de Castro Pereira, Inara Caroline Marcelino Martins, Cecília Alves Macedo, Joyce Noelly Vitor Santos, Elisângela Andrade Assis Madeira, Vanessa Gomes Brandão Rodrigues, Maria Cecília Sales Mendes Prates, Frederico Lopes Alves, Emílio Henrique Barroso Maciel, Vanessa Pereira Lima, Henrique Silveira Costa, Vanessa Amaral Mendonça, Pedro Henrique Scheidt Figueiredo

**Affiliations:** 1 Universidade Federal dos Vales do Jequitinhonha e Mucuri, Programa de Pós-Graduação em Reabilitação e Desempenho Funcional, Diamantina, MG, Brazil.; 2 Universidade Federal dos Vales do Jequitinhonha e Mucuri, Departamento de Fisioterapia, Diamantina, MG, Brazil.; 3 Universidade Federal dos Vales do Jequitinhonha e Mucuri, Programa de Pós-Graduação em Ciências da Saúde, Diamantina, MG, Brazil.; 4 Santa Casa de Caridade de Diamantina, Setor de Hemodiálise, Diamantina, MG, Brazil.

**Keywords:** Músculos Respiratórios, Tolerância ao Exercício, Força Muscular, Qualidade de Vida, Hemodiálise, Desenvolvimento Sustentável, ODS-3.

## Abstract

**Introdução:**

Pacientes em hemodiálise frequentemente apresentam fraqueza muscular inspiratória, a qual se associa ao declínio da qualidade de vida relacionada à saúde (QVRS). Contudo, permanece desconhecido o quanto dessa associação é mediada pela aptidão cardiorrespiratória.

**Objetivo:**

Avaliar o efeito mediador da aptidão cardiorrespiratória na associação entre força muscular inspiratória e QVRS em pessoas em hemodiálise.

**Métodos:**

Estudo transversal, no qual pessoas em hemodiálise foram avaliadas quanto à força muscular inspiratória, pela mensuração da pressão inspiratória máxima (PImáx); à aptidão cardiorrespiratória, pelo *Incremental Shuttle Walk Test* (ISWT); e à QVRS, pelos componentes físico (CFS), mental (CMS) e geral (CGS) sumarizados do questionário *Kidney Disease Quality of Life*. Análises de mediação simples foram realizadas usando a macro PROCESS, versão 3.5 para SPSS, tendo QVRS como variável dependente, PImáx como variável independente e aptidão cardiorrespiratória como mediadora.

**Resultados:**

Foram avaliados 64 indivíduos, 67,2% homens, com média de idade de 48,1 anos (IC95%: 44,3–51,9). A aptidão cardiorrespiratória exerceu efeito mediador parcial na associação entre PImáx e CFS (β = 0,14; IC95%: 0,02–0,27), resultando em uma redução de 6% da variância do CFS explicada pela PImáx (6% do *R* ^2^). A proporção de mediação foi de 32,5%. Não houve associação entre PImáx e CMS, e a aptidão cardiorrespiratória não apresentou efeito mediador na relação PImáx e CGS (β = 0,13; p = 0,350).

**Conclusão:**

Na amostra estudada, a associação entre força muscular inspiratória e aspectos físicos da QVRS é parcialmente mediada pela aptidão cardiorrespiratória. No entanto, esse efeito mediador não foi observado para aspectos mentais, tampouco para aspectos gerais da QVRS.

## Introdução

A doença renal crônica (DRC) consiste na redução progressiva e irreversível da função renal, ocasionada pela presença de lesão nos rins, por um período superior a três meses, com impacto na saúde^1^. Com o avanço da doença, o sistema renal torna-se incapaz de manter a homeostase. Nessa fase, indica-se o tratamento por terapia renal substitutiva^1^, sendo a hemodiálise a modalidade de terapia mais utilizada^2^. De acordo com o Censo Brasileiro de Diálise, no ano de 2023^3^, o número de pacientes em diálise foi, em média, 157.357, as taxas de prevalência foram de 771 por milhão da população, e a incidência, de 251 casos por milhão da população.

Embora seja um procedimento fundamental para o controle dos sintomas e o aumento da sobrevida dos pacientes acometidos, o tratamento por hemodiálise, assim como a própria DRC, leva a alterações de estrutura e função muscular, com consequente redução da aptidão cardiorrespiratória^4,5^. Uma vez presente, a redução da aptidão cardiorrespiratória pode contribuir para limitações em atividades que necessitam de esforço físico, afetando o nível de atividade de vida diária e aspectos emocionais, com consequente declínio da qualidade de vida relacionada à saúde (QVRS)^6^. Adicionalmente, a redução da aptidão cardiorrespiratória está associada a um pior prognóstico nessa população^7,8^.

As alterações da estrutura e função muscular em pacientes em hemodiálise podem também afetar a função dos músculos inspiratórios, manifestada pela redução da pressão inspiratória máxima (PImáx)^9,10^. A redução da PImáx tem sido apontada como um preditor independente de comprometimento da aptidão cardiorrespiratória nessa população^11^, além de estar associada ao declínio da QVRS^12^. A relação entre força muscular inspiratória e QVRS tem sido também inferida por meio dos desfechos positivos do treinamento específico da musculatura inspiratória^11,13,14^.

Portanto, a associação entre a PImáx e a aptidão cardiorrespiratória em pessoas com DRC em tratamento por hemodiálise já está descrita na literatura, assim como sua associação com a QVRS. No entanto, não se sabe se a força muscular inspiratória exerce um efeito direto sobre a QVRS ou se essa associação reflete apenas uma influência indireta, mediada pela redução da aptidão cardiorrespiratória. A elucidação dessa questão pode auxiliar na compreensão dos mecanismos envolvidos na relação entre força muscular inspiratória e QVRS. O objetivo do presente estudo foi avaliar o efeito mediador da aptidão cardiorrespiratória na associação entre força muscular inspiratória e QVRS em pessoas em hemodiálise.

## Métodos

### Desenho do Estudo

Trata-se de um estudo transversal, realizado no setor de Hemodiálise da Santa Casa de Caridade de Diamantina–MG, em parceria com o Laboratório de Reabilitação Cardiovascular (LABCAR) da Universidade Federal dos Vales do Jequitinhonha e Mucuri (UFVJM, Diamantina-MG, Brasil). Esta pesquisa foi aprovada pelo Comitê de Ética Institucional (CAAE 60169822.10000.5108) e todos os participantes concordaram em participar do estudo por meio da assinatura do Termo de Consentimento Livre e Esclarecido.

### Participantes

De maio a agosto de 2023, foram avaliados indivíduos com DRC, submetidos a tratamento por hemodiálise por um período superior a três meses e com frequência semanal de três vezes por semana, com idade superior a 18 anos, de ambos os sexos. Foram excluídos os indivíduos com pressão arterial sistólica e diastólica de repouso superiores a 180 e/ou 110 mmHg, respectivamente^15^; com anemia grave (hemoglobina < 8 g/dL)^16^; angina instável; arritmias ventriculares complexas; doença metabólica grave; infarto agudo do miocárdio há menos de um mês; afecções agudas; aneurisma de aorta; estenose aórtica grave; comprometimento respiratório, neurológico ou musculoesquelético que contraindicasse a prática de atividade física; ou incapacidade de executar o protocolo de avaliação.

O cálculo amostral foi realizado no software G*Power, versão 3.1 (Franz Faul, *Universität Kiel*, Alemanha). Estimou-se uma amostra de 50 participantes, considerando um R^2^ ajustado de 0,23^12^ entre a PImáx e o componente físico sumarizado do questionário *Kidney Disease Quality of Life* (KDQOL-SF)^17^, poder estatístico de 80%, erro alfa de 5% e cinco preditores.

### Procedimentos

As avaliações ocorreram ao longo de uma semana, imediatamente antes das sessões de hemodiálise, na seguinte sequência^18^: imediatamente antes da primeira sessão semanal – força muscular inspiratória; imediatamente antes da segunda sessão semanal – QVRS; e imediatamente antes da terceira sessão semanal – aptidão cardiorrespiratória.

### Força muscular inspiratória

Foi avaliada por meio da mensuração da PImáx, utilizando um manômetro analógico (MV-300/300, Ger-Ar, São Paulo, Brasil), calibrado, com variação de -300 a 300 cmH_2_O e precisão de 10 cmH_2_O. O aparelho foi acoplado à boca do voluntário por meio de uma traqueia plástica de 14 cm de comprimento e 2 cm de diâmetro, conectada a um bucal contendo um orifício de 2 mm na face superior para compensar a variação de pressão provocada pela musculatura da orofaringe^19^. Para a avaliação, o indivíduo permaneceu em repouso na posição sentada durante 5 minutos, com o dorso apoiado no encosto da cadeira e os pés apoiados no chão, com flexão de 90º de quadril e joelhos. Em seguida, foi orientado a realizar uma inspiração com a maior força possível, com as narinas ocluídas por um clipe, a partir do volume residual. As incursões inspiratórias foram repetidas para aprendizado e, em seguida, foram realizadas três medidas tecnicamente satisfatórias, com repouso de 60 segundos entre elas. Foram consideradas satisfatórias as medidas que apresentaram variação inferior a 10%, sem a presença de escape aéreo, sustentadas por, pelo menos, um segundo. O valor considerado para fins de análise foi o mais alto, desde que não tivesse sido obtido na última manobra. Estímulos verbais padronizados foram oferecidos durante as incursões inspiratórias máximas^12,20^. A fraqueza muscular inspiratória foi considerada quando os valores de PImáx estavam abaixo dos limites inferiores da normalidade, conforme valores preditos para a população brasileira saudável^21^.

### Aptidão cardiorrespiratória

A aptidão cardiorrespiratória foi avaliada por meio do *Incremental Shuttle Walk Test* (ISWT), realizado em um corredor de 10 m, delimitado por dois cones^22^. Foi aplicado o teste de 12 níveis. Os participantes foram orientados a realizar o percurso de acordo com a emissão do sinal sonoro e não receberam nenhum tipo de incentivo dos avaliadores. O teste foi finalizado quando o indivíduo não atingiu o final do percurso antes da emissão do sinal sonoro – que indicaria a troca de sentido – por duas vezes consecutivas^11,23^.

### Qualidade de vida relacionada à saúde – QVRS

O questionário utilizado para avaliar a QVRS foi o KDQOL-SF^17^, específico para a população com DRC, com adaptação e validação para a população brasileira^24^. Esse instrumento é composto por 24 perguntas multidimensionais, com 43 itens específicos para DRC, 36 itens de abordagem genérica e um item de identificação da condição de saúde. Os itens presentes no KDQOL-SF são agrupados em 19 domínios, dentre eles, 11 domínios específicos para DRC dialítica e oito domínios genéricos. Os domínios são pontuados com um escore final que varia de 0 (pior estado de saúde) a 100 (melhor estado de saúde). Domínios genéricos foram utilizados para construir o componente físico sumarizado (CFS) e o componente mental sumarizado (CMS). Domínios específicos foram utilizados para construir o componente geral sumarizado (CGS)^17^. Os componentes sumarizados foram as variáveis de interesse analisadas.

### Análise Estatística

A análise dos dados foi realizada por meio do software SPSS, versão 25.0. Os dados foram descritos por frequência absoluta e relativa para as variáveis categóricas e por média ± desvio padrão para as variáveis quantitativas contínuas. Análises de correlação foram realizadas pelos coeficientes de *Pearson* ou *Spearman*, conforme o teste de normalidade (*Kolmogorov–Smirnov*). Correlações muito fracas foram consideradas quando r < 0,31 ou > −0,31; fracas, quando r estava entre 0,31 e 0,50 ou entre −0,50 e −0,31; moderadas, quando r estava entre 0,51 e 0,70 ou entre −0,70 e −0,51; e fortes, quando r > 0,71 ou < −0,71^25^. A significância estatística foi considerada quando p < 0,05.

Para testar as hipóteses sobre os efeitos de mediação da aptidão cardiorrespiratória na relação entre PImáx e QVRS, foi utilizada a macro PROCESS, versão 3.5, para o SPSS, com procedimento de *bootstrapping* de 5.000 amostras para estimar o intervalo de confiança de 95% (IC95%). Nos modelos de mediação simples, são verificadas as relações entre três variáveis: a independente (PImáx), a mediadora (aptidão cardiorrespiratória) e a dependente (QVRS). Assim, são avaliados o efeito da variável independente sobre a variável mediadora (PImáx → aptidão cardiorrespiratória), o efeito da variável mediadora sobre a variável dependente (aptidão cardiorrespiratória → QVRS) e os efeitos total, direto e indireto da variável independente (PImáx) sobre a variável dependente (QVRS).

O efeito total refere-se ao impacto da variável independente sobre a variável dependente (PImáx → QVRS). O efeito direto é o impacto da variável independente sobre a variável dependente, com o mediador inserido na análise (PImáx → QVRS, controlado pela aptidão cardiorrespiratória). Já o efeito indireto é o impacto da variável indepen­dente sobre a variável dependente, passando pelo mediador (PImáx → aptidão cardiorrespiratória → QVRS). Quando o efeito indireto é estatisticamente significativo, indica que houve mediação da aptidão cardiorrespiratória na relação entre PImáx → QVRS.

O efeito mediador pode ser completo ou parcial. Na mediação completa, o efeito direto deixa de ser significativo após a inserção do mediador (aptidão cardiorrespiratória). Já na mediação parcial, ocorre uma redução na magnitude da regressão e uma redução no coeficiente do efeito direto, quando comparado com o coeficiente do efeito total, mas sem perder a significância (p < 0,05). Nessa última situação, a aptidão cardiorrespiratória exerce um efeito mediador parcial sobre a associação entre a PImáx e a QVRS. Em outras palavras, a relação entre a PImáx e a QVRS é parcialmente mediada pela aptidão cardiorrespiratória^26^. Além disso, pode ser calculada a proporção de mediação, que representa a força da mediação, ou o quanto a aptidão cardiorrespiratória explica a associação entre PImáx e QVRS^26^. A proporção de mediação foi obtida por meio do cálculo da razão entre o efeito indireto e o efeito total^26^.

## Resultados

De 115 pacientes em hemodiálise, 101 foram considerados elegíveis, e 64 indivíduos foram incluídos no estudo (Figura 1). As características da amostra estão apresentadas na Tabela 1. Os indivíduos eram, predominantemente, do sexo masculino, com média de idade de 48,1 anos (IC95%: 44,3–51,9) e eutróficos. A comorbidade mais prevalente foi a hipertensão arterial sistêmica (82,8%). A maioria dos participantes fazia uso do medicamento eritropoietina (90,6%). Fraqueza muscular inspiratória foi observada em 21 indivíduos (32,8%).

**Figura 1:** Fluxograma.

**Tabela 1:** CARACTERÍSTICAS DA AMOSTRA (N = **64**)

Variáveis	Valores
Sexo, n (%)	
Masculino	43 (67,2)
Feminino	21 (32,8)
Idade (anos)	48,1 ± 15,1
IMC (Kg/m2)	24,1 ± 5,1
Tempo de tratamento (anos)	4,7 ± 4,2
Bioquímica	
Kt/V	1,5 ± 0,3
Hemoglobina (mg/dL)	10,6 ± 1,9
Hematócrito (%)	33,8 ± 4,6
Força muscular inspiratória	
PImáx (cmH2O)	-90,1 ± 26,3
Fraqueza muscular inspiratória, n (%)	21 (32,8%)
ISWT	
Distância (m)	419,7 ± 224,4

Abreviaturas – IMC: Índice de Massa Corporal; Kt/V: Índice de Efetividade do Tratamento Hemodialítico; PImáx: Pressão Inspiratória Máxima; ISWT: *Incremental Shuttle Walk Test*.

Nota – Dados apresentados como média ± desvio padrão ou n (%).

Na Tabela 2, estão apresentados os escores dos domínios da QVRS. Os componentes sumarizados mais afetados foram os componentes físico e mental. Observou-se uma correlação fraca, significativa, entre a PImáx e os componentes físico e geral sumarizados do KDQOL-SF (r = 0,40 e p = 0,001; r = 0,43 e p < 0,001, respectivamente). Não foi observada correlação significativa com o CMS (r = 0,11 e p = 0,38). A distância percorrida no ISWT apresentou correlação fraca, significativa, com o CFS (r = 0,38 e p = 0,002) e correlação moderada com a PImáx (r = 0,58 e p < 0,001). Não foram encontradas correlações significativas entre a distância do ISWT e os componentes geral e mental sumarizados (r = 0,27 e p = 0,29; r = 0,98 e p = 0,44, respectivamente). Com base nas corre­lações observadas, as análises de mediação foram realizadas utilizando como variáveis dependentes apenas o CFS e o CGS.

**Tabela 2:** DOMÍNIOS DA QUALIDADE DE VIDA RELACIONADA À SAÚDE PELO QUESTIONÁRIO **KDQOL-SF**

Variáveis	Valores
Dimensão Específica	
Sintomas	85,1 ± 13,6
Efeitos da DRC	77,2 ± 17,8
Carga da DRC	44,7 ± 30,8
Trabalho	26,3 ± 39,7
Cognição	90,2 ± 15,0
Interação Social	88,0 ± 22,2
Função Sexual	50,3 ± 49,5
Sono	76,1 ± 24,9
Suporte Social	84,1 ± 29,1
Encorajamento pela Equipe de Saúde	83,3 ± 30,4
Satisfação com o Tratamento	80,9 ± 20,3
CGS	71,5 ± 12,1
Dimensão Genérica	
Capacidade Funcional	75,2 ± 26,4
Função Física	62,0 ± 33,3
Dor	73,6 ± 26,1
Estado Geral de Saúde	55,1 ± 27,1
Bem-Estar Emocional	66,6 ± 40,2
Papel Emocional	81,3 ± 22,8
Função Social	88,0 ± 22,2
Energia/Fadiga	70,8 ± 25,5
CFS	46,4 ± 10,3
CMS	44,9 ± 20,7

Abreviaturas – KDQOL-SF: *Kidney Disease Quality of Life*; DRC: Doença Renal Crônica; CFS: Componente Físico Sumarizado; CMS: Componente Mental Sumarizado; CGS: Componente Geral Sumarizado.

Nota – Dados apresentados como média ± desvio padrão.

Os resultados das análises de mediação entre PImáx e CFS estão apresentados no Arquivo Suplementar 1 e na Tabela 3. Foi observado um efeito de mediação parcial significativo da aptidão cardiorrespiratória na relação entre PImáx e CFS (β = 0,14; IC95%: 0,02–0,27). A inserção da aptidão cardiorrespiratória como variável media­dora resultou em uma redução de 6% na variância dos escores do CFS explicada pela PImáx (redução de 6% no R^2^). O percentual de mediação atingiu 32,5%. A aptidão cardiorrespiratória não apres­entou efeito mediador sobre a relação entre PImáx e CGS (β = 0,13; p = 0,350), conforme apresentado no Arquivo Suplementar 2 e na Tabela 4.

**Tabela 3:** ANÁLISE DE MEDIAÇÃO DA APTIDÃO CARDIORRESPIRATÓRIA PARA A RELAÇÃO ENTRE A PRESSÃO INSPIRATÓRIA MÁXIMA E A QUALIDADE DE VIDA RELACIONADA À SAÚDE (COMPONENTE FÍSICO SUMARIZADO)

Efeitos das variáveis	R^2^	β	p
Efeito da variável mediadora na variável dependente	0,17	0,28	0,033
Efeito da variável independente na variável mediadora	0,24	0,50	< 0,001
Efeito total da variável independente na variável dependente	0,19	0,43	< 0,001
Efeito direto da variável independente na variável dependente (com inclusão da variável mediadora)	0,25	0,29	0,028
	β	IC95%	p
Efeito indireto da variável independente na variável dependente	0,14	0,02–0,27	0,028

Notas – Variável independente: pressão inspiratória máxima; Variável dependente: componente físico sumarizado da qualidade de vida relacionada à saúde; Variável mediadora: aptidão cardiorrespiratória; R2: coeficiente de determinação; β: coeficiente padronizado; IC95%: intervalo de confiança de 95%.

**Tabela 4:** ANÁLISE DE MEDIAÇÃO DA APTIDÃO CARDIORRESPIRATÓRIA PARA A RELAÇÃO ENTRE A PRESSÃO INSPIRATÓRIA MÁXIMA E A QUALIDADE DE VIDA RELACIONADA À SAÚDE (COMPONENTE GERAL SUMARIZADO)

Efeitos das variáveis	R^2^	β	p
Efeito da variável mediadora na variável dependente	0,07	0,13	0,350
Efeito da variável independente na variável mediadora	0,16	0,50	< 0,001
Efeito total da variável independente na variável dependente	0,15	0,40	< 0,001
Efeito direto da variável independente na variável dependente (com inclusão da variável mediadora)	0,18	0,34	0,013
	β	IC95%	p
Efeito indireto da variável independente na variável dependente	–	–	–

Notas – Variável independente: pressão inspiratória máxima; Variável dependente: componente geral sumarizado da qualidade de vida relacionada à saúde; Variável mediadora: aptidão cardiorrespiratória; R2: coeficiente de determinação; β: coeficiente padronizado; IC95%: intervalo de confiança de 95%.

## Discussão

O presente estudo teve como objetivo avaliar o efeito mediador da aptidão cardiorrespiratória na associação entre PImáx e qualidade de vida relacionada à saúde (QVRS). Até onde se tem conhecimento, este é o primeiro estudo a abordar especificamente essa questão, e os principais achados foram: 1) a presença de uma associação direta entre a PImáx e a QVRS; e 2) a relação entre a PImáx e o componente físico sumarizado da QVRS ser parcialmente mediada pela aptidão cardiorrespiratória. Esses achados têm importante relevância clínica, por evidenciarem a influência da PImáx sobre a QVRS e o efeito mediador da aptidão cardiorrespiratória nessa relação.

Já é bem estabelecido na literatura que a aptidão cardiorrespiratória é comprometida em pessoas em hemodiálise, devido aos efeitos do curso natural da doença, do próprio tratamento dialítico e das mudanças no estilo de vida decorrentes do tratamento, resultando em restrições na participação social, repercutindo na QVRS^^4,5,6,27^^. Assim, as limitações para a realização das atividades de vida diária e as restrições na participação social e no lazer, determinadas pelo conjunto de sinais e sintomas clínicos, favorecem o declínio da QVRS^27^. O nosso estudo vai ao encontro dessa afirmativa, pois também identificou a associação da aptidão cardiorrespiratória com a QVRS, principalmente nos componentes físicos do questionário KDQOL-SF.

Na primeira análise realizada no presente estudo, foi encontrada uma associação significativa entre a PImáx e os componentes físicos e gerais sumarizados do KDQOL-SF, ressaltando a influência da força muscular inspiratória na QVRS dos pacientes em hemodiálise. Esse resultado corrobora os achados de Vieira *et al.* (2020)^12^, que apresentaram a PImáx como um preditor da QVRS, independentemente do tempo de tratamento por hemodiálise, da idade, de comorbidades associadas e da força muscular dos membros inferiores.

Além da associação com a QVRS, a PImáx é apontada como um fator determinante da aptidão cardiorrespiratória em pacientes em hemodiálise^10^. Assim, a presença de fraqueza muscular inspiratória pode afetar negativamente a aptidão cardiorrespiratória, o que contribui para o declínio da QVRS^11,12^. Na presente investigação, foi constatado que a associação entre a PImáx e a QVRS possui um efeito mediado pela aptidão cardiorrespiratória. Isso sugere que, na presença de menor força muscular inspiratória, a capacidade ventilatória pode ser afetada, provocando uma redução na aptidão cardiorrespiratória e, consequentemente, afetando os aspectos físicos da QVRS.

Entretanto, um resultado relevante do presente trabalho foi a associação da PImáx com os aspectos gerais da QVRS, sem mediação da aptidão cardiorrespiratória. Essa associação direta sugere que a PImáx influencia a QVRS por vias que não dependem exclusivamente da aptidão cardiorrespiratória. Embora relações entre força inspiratória, aptidão cardiorrespiratória e QVRS já tenham sido descritas em pessoas em hemodiálise^10,12,28^, não foram identificados estudos que investigassem se a PImáx se associa à QVRS de forma independente da aptidão cardiorrespiratória, o que limita a realização de comparações diretas com os nossos achados.

A QVRS de pessoas em hemodiálise é multidimensional^29^, sendo fortemente determinada pela carga de sintomas^30^. Campos *et al*. (2024)^31^ demonstraram que sintomas limitantes, como a sensação de dispneia, cansaço e fraqueza, foram relatados como fatores importantes para a QVRS em uma amostra de pessoas em hemodiálise. A fraqueza muscular inspiratória pode refletir alterações sistêmicas de estrutura e de função muscular, comuns nessa população, indicando um pior estado nutricional ou menor reserva muscular^32^. Condições estas, conhecidamente associadas à manifestação de sintomas e à pior percepção de qualidade de vida^29^.

Além disso, considerando que o aumento do trabalho da musculatura respiratória é um mecanismo associado à manifestação da dispneia^33^ e do cansaço^34^, espera-se que a redução da PImáx predisponha ao aumento do trabalho respiratório e à manifestação de sintomas, limitando a execução de atividades cotidianas. Esses mecanismos fisiológicos estão em consonância com achados observacionais prévios, como os de Vieira *et al*. (2020)^12^, que relataram correlação significativa entre a PImáx e o domínio “sintomas” do KDQOL (r = 0,45; p < 0,001). No entanto, ressalta-se que os mecanismos envolvidos na relação direta entre a força inspiratória e os aspectos gerais da QVRS nessa população necessitam ser elucidados em estudos futuros.

A associação da PImáx com a aptidão cardiorrespiratória e a QVRS pode também ser inferida indiretamente a partir de dados da literatura, que apontam efeitos benéficos do treinamento muscular inspiratório nessa população, não apenas sobre a força dos músculos respiratórios, mas também sobre a aptidão cardiorrespiratória e a QVRS^^11,12,13^^. Portanto, pode-se inferir que a melhora da força muscular inspiratória afeta a QVRS de forma direta e, indiretamente, por meio do aumento da aptidão cardiorrespiratória^27,35^.

Além dos benefícios do treinamento muscular inspiratório, estudos demonstraram que o treinamento físico tradicional, realizado durante a sessão de hemodiálise, promoveu melhoras na aptidão cardiorrespiratória, bem como na força e função dos músculos inspiratórios^32^. Esse achado sugere que intervenções baseadas em exercícios físicos tradicionais, capazes de melhorar a força muscular inspiratória e a aptidão cardiorrespiratória, podem influenciar positivamente a QVRS nessa população.

O presente estudo demonstrou, usando uma metodologia criteriosa, o papel mediador da capacidade de exercício na relação entre a PImáx e os aspectos físicos da QVRS e a associação da PImáx com os aspectos gerais da QVRS, sem efeito mediado pela capacidade de exercício. Esses achados são clinicamente relevantes, pois apontam para a importância de estratégias de prevenção e reabilitação, a fim de manter ou aprimorar a função da musculatura inspiratória, por meio de abordagens nutricionais e de treinamento muscular específico. Tais estratégias podem amenizar o declínio ou melhorar a QVRS da população em hemodiálise. Entretanto, estudos de intervenção ainda são necessários para confirmar essa hipótese.

Como limitações, este estudo apresenta uma amostra de indivíduos, em sua maioria, com menor idade e menor número de comorbidades associadas, quando comparado a outros estudos. Assim, a extrapolação dos resultados para populações com características clínicas e demográficas diferentes deve ser avaliada com cautela.

Em conclusão, em pessoas em hemodiálise, a associação entre força muscular inspiratória e aspectos físicos da QVRS é parcialmente mediada pela aptidão cardiorrespiratória. Entretanto, esse efeito mediador não foi observado para aspectos mentais, tampouco para aspectos gerais da QVRS.

## Disponibilidade de Dados

Os conjuntos de dados gerados e/ou analisados durante o presente estudo estão disponíveis mediante solicitação razoável ao autor correspondente.

## Material Suplementar

O seguinte material online está disponível para o presente artigo:

Arquivo suplementar 1 - Efeito mediador da aptidão cardiorrespiratória na relação entre a PImáx e o componente físico sumarizado da qualidade de vida relacionada à saúde.

Arquivo suplementar 2 - Efeito mediador da aptidão cardiorrespiratória na relação entre a PImáx e o componente geral sumarizado da qualidade de vida relacionada à saúde.
